# Supplementary material for: Genome-scale Capture C promoter interactions implicate effector genes at GWAS loci for bone mineral density
Source: Nat Commun. 2019 Mar 19;10:1260. doi: 10.1038/s41467-019-09302-x (PMC6425012; doi:10.1038/s41467-019-09302-x)
Supplement: Supplementary file 1 — Supplementary Information [file 41467_2019_9302_MOESM1_ESM.pdf]

SUPPLEMENTARY INFORMATION

Genome-scale Capture C promoter interactions implicate effector genes at GWAS loci for bone mineral density

Chesi et al.

SUPPLEMENTARY INFORMATION

Capture C coverage tracks in bigWig format for specific baits (for visualization in the UCSC Genome Browser) can be obtained using the following script, where hicup.bam is the output of the HiCUP pipeline and bait.bed contains the coordinates of the bait of interest in bed format.

```
module load bedtools
perl get_captured_reads.pl --baits bait.bed hicup.bam
bamToBed -i hicup.captured.bam > hicup.capture.bed
subtractBed -a hicup.capture.bed -b bait.bed > hicup.captures.bed
fetchChromSizes hg19 > hg19.chrom.sizes
sortBed -i hicup.captures.bed | genomeCoverageBed -i - -bg -g hg19.chrom.sizes >
bait.captures.bedGraph
bedGraphToBigWig bait.captures.bedGraph hg19.chrom.sizes bait.bw
```

The BEDTools suite is available at https://bedtools.readthedocs.io/en/latest/, get_captured_reads.pl is a perl script provided as part of the HiCUP pipeline (https://www.bioinformatics.babraham.ac.uk/projects/hicup/) and fetchChromSizes is available from the UCSC team at http://hgdownload.cse.ucsc.edu/admin/exe/linux.x86_64/fetchChromSizes.

Supplementary Table 1

A. Cell lines information

Sample No.	Cell line name	Sex	Age (Y)	Experiments			
1	7043	M	28.8		ATAC-seq (1 rep)		
2	7078	M	24.6			RNA-seq	siRNA targeting (SFRP4, EPDR1, ING3, CPED1, WNT16)
3	8004	M	22	Capture C	ATAC-seq (3 rep)	RNA-seq	siRNA targeting (WNT16)
4	8013	M	22	Capture C	ATAC-seq (3 rep)	RNA-seq	siRNA targeting (SFRP4, EPDR1, ING3, CPED1, WNT16)
5	080454B (Lonza2)	M	20	Capture C	ATAC-seq (2 rep)		siRNA targeting (SFRP4, EPDR1, ING3, CPED1, WNT16)
6	8001R	F	26				siRNA targeting (SFRP4, EPDR1)

B. qPCR primer sets

Name	Sequence
hGAPDH_S	TGG TAT CGT GGA AGG ACT CAT GAC
hGAPDH_AS	ATG CCA GTG AGC TTC CCG TTC AGC
hID1_S	CTC TAC GAC ATG AAC GGC TGT
hID1_AS	TGC TCA CCT TGC GGT TCT G
hALP_S	GTA AGG ACA TCG CCT ACC AG
hALP_AS	GGC TTT CTC GTC ACT CTC AT
hOSX_S	GCT TGA GGA GGA AGT TCA CTA T
hOSX_AS	GCT TCT TTG TGC CTG CTT TG
hRunx2_S	CAG ACC AGC AGC ACT CCA TA
hRunx2_AS	CAG CGT CAA CAC CAT CAT TC
hSFRP4_S	CTG TCT ATG ACC GTG GCG TG
hSFRP4_AS	TGC CAA AGT TGG CTT CAC CT
hEPDR1_S	CGC AGC AGT GGG AGG G
hEPDR1_AS	CTT TGA GCA CTG CTT GGT GG
hING3_S	GCT GGA TCA GGA ACT GGC TAA
hING3_AS	ACT GGC TGT GAA GGA GTG TC
hCPED1_S	GCC CCA CAA CTG CCA ATA TG
hCPED1_AS	AGT GCC ATG TAC TTT CTG CCA
hWNT16_S	GTG CGG GAG CCA GTT CAG
hWNT16_AS	CCT GCA GCC ATC ACA GCA TA
hCEBPa_S	GAT AAC CTT GTG CCT TGG AAA TG
hCEBPa_AS	GAG GCA GGA AAC CTC CAA ATA
hPPARg_S	ATG TCG TGT CTG TGG AGA TAA
hPPARg_AS	CCA AAC CTG ATG GCA TTA TGA G

C. ON-TARGETplus siRNA

Catalog Number	Name	Target Sequence1	Target Sequence 2	Target Sequence 3	Target Sequence 4	mRNA Accession Number
LQ-011388-00-0002	ON-TARGETplus Human SFRP4	GGAGAGAUCAGCUUAGUAA	CAACGGUGGUGGAUGUAAA	GGAGGAUGUUAAGUGGAUA	GAGCUAACUAGUUUCCAAA	NM_003014.3
LQ-013378-01-0002	ON-TARGETplus Human EPDR1	CUACACUGGCAUAGCACGA	GGACUAGAGUUCCCUCGUA	CUGGAGAAGAUGAGCGAAG	GAAGAAAAUUGAAUGGCGA	NM_001242946.1, NM_001242948.1, NM_017549.4
LQ-007000-00-0002	ON-TARGETplus Human ING3	CUAUAAAGCUUUGGAAGAU	UGGCAAACCAGAUAUAUGA	CAACUAGAACAAAGAGUCA	GGGAAGAGCAAAUGGCAUC	NM_019071.2, NM_198267.1
LQ-018816-02-00002	ON-TARGETplus Human CPED1	GCUGAUUCAUCCAGAGGAA	UGUGAUUCUUAAAGCGUAU	CUCAAUAUUUGUUGUGGAU	UGCCUUAGUUGUUCGGACA	NM_001105533.1, NM_024913.4
LQ-010821-00-0002	ON-TARGETplus Human WNT16	CCAAGUUGAUGUCAGUAGA	CCGAUGAUGUCCAGUAUGG	GGCAGAGAAUGCAACCGUA	GCAGAAAGUUCCUAGAUUU	NM_001105533.1, NM_024913.4

Supplementary Table 2

Summary of Capture C interactions detected by CHiCAGO

Cell Type	Res	Bait Frags	Bait Frags with Interactions	Interactions	Trans Interactions	Bait-to-bait	Median bait-PIR distance (bp)	Unique non-bait PIR	non-bait PIR interacting with a single bait frags	non-bait PIR interacting with >=4 bait frags
osteoblasts	1-frag	36,691	25,265 (69.0%)	168,721	1,684 (1%)	15,285 (9%)	14,934	133,159	116,911 (88%)	689 (0.5%)
	4-frag	34,354	26,310 (77.0%)	215,992	1,416 (1%)	36,343 (17%)	105,174	121,829	86,931 (71%)	5,302 (4%)
	merged			295,422	2,076 (1%)	41,459 (14%)	50,459	209,370	176,241 (84%)	2,295 (1%)
HepG2	1-frag	36,691	22,377 (61.0%)	65,523	1,007 (1.5%)	10,304 (15.7%)	9,846	49,253	44,693 (90.74%)	296 (0.60%)
	4-frag	34,354	23,633 (68.8%)	107,644	858 (0.80%)	29,619 (27.5%)	72,417	55,566	42,344 (76.20%)	2,167 (3.90%)
	merged			135,034	1,353 (1.00%)	33,546 (24.8%)	42,223	84,720	72,909 (86.06%)	1,072 (1.27%)

Supplementary Table 3

ENCODE histone marks tracks from primary human osteoblasts

Cell Type	Species	ChIP antibody Target	GEO Accession	File Type	Lab
osteoblasts	Homo Sapiens	CTCF	GSM733784	broadPeak.bed	Bernstein - Broad Institute
osteoblasts	Homo Sapiens	H3K27ac	GSM733739	broadPeak.bed	Bernstein - Broad Institute
osteoblasts	Homo Sapiens	H3K27me3	GSM1003466	broadPeak.bed	Bernstein - Broad Institute
osteoblasts	Homo Sapiens	H3K36me3	GSM733721	broadPeak.bed	Bernstein - Broad Institute
osteoblasts	Homo Sapiens	H3K4me1	GSM733704	broadPeak.bed	Bernstein - Broad Institute
osteoblasts	Homo Sapiens	H3K4me2	GSM733779	broadPeak.bed	Bernstein - Broad Institute
osteoblasts	Homo Sapiens	H3K4me3	GSM1003506	broadPeak.bed	Bernstein - Broad Institute
osteoblasts	Homo Sapiens	H3K9me3	GSM733681	broadPeak.bed	Bernstein - Broad Institute

Supplementary Figure 1

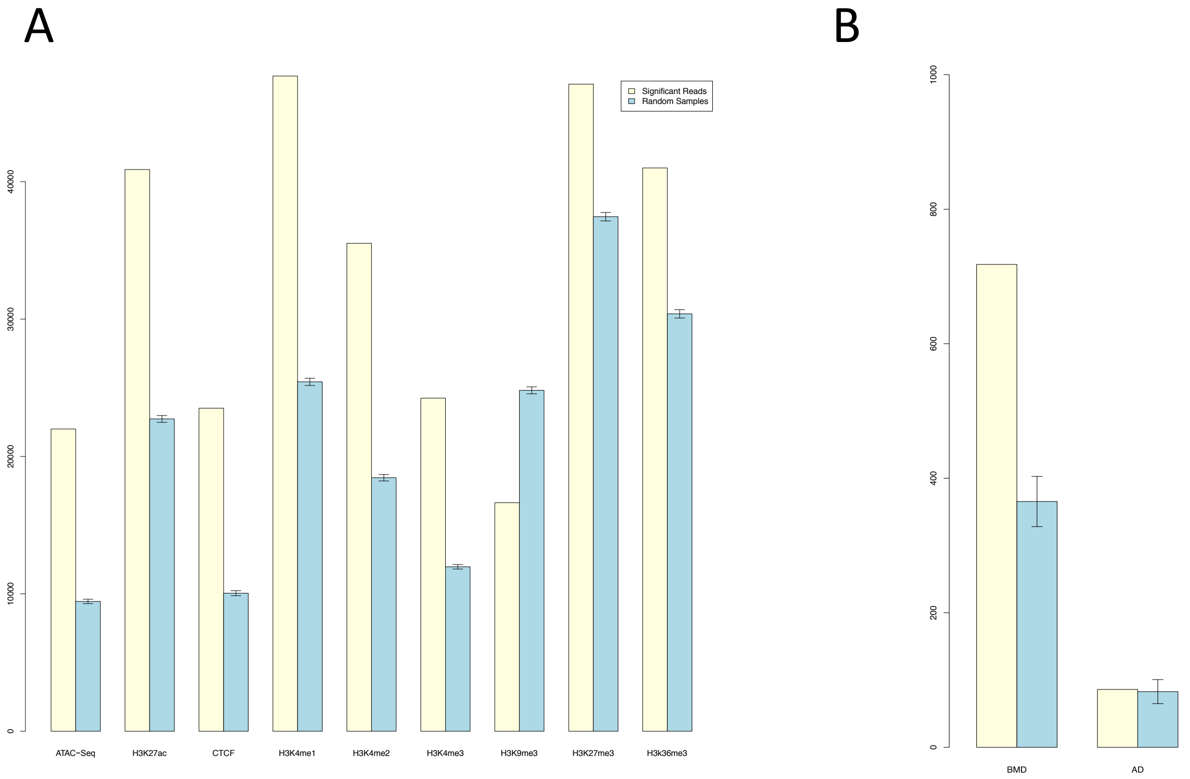

Promoter-interacting regions (PIR) enrichment. (A) Enrichment of PIR in chromatin marks. Yellow bars: number of overlaps with significant (CHiCAGO score>5) cis-interacting fragments (at 4-fragment resolution; bait-to-bait interactions were excluded); blue bars: expected overlaps based on 100 random subsets of fragments with a similar distribution of distances from the baits. ATAC-seq data is from our own experiments in hMSC-derived osteoblasts; other markers are from primary human osteoblasts from the ENCODE project. Error bars represent 95% confidence intervals. (B) Enrichment of PIR in BMD GWAS signals. As above, but overlaps are computed with BMD or Alzheimer’s disease GWAS SNPs and their proxies (r^2>0.4).

Supplementary Figure 2

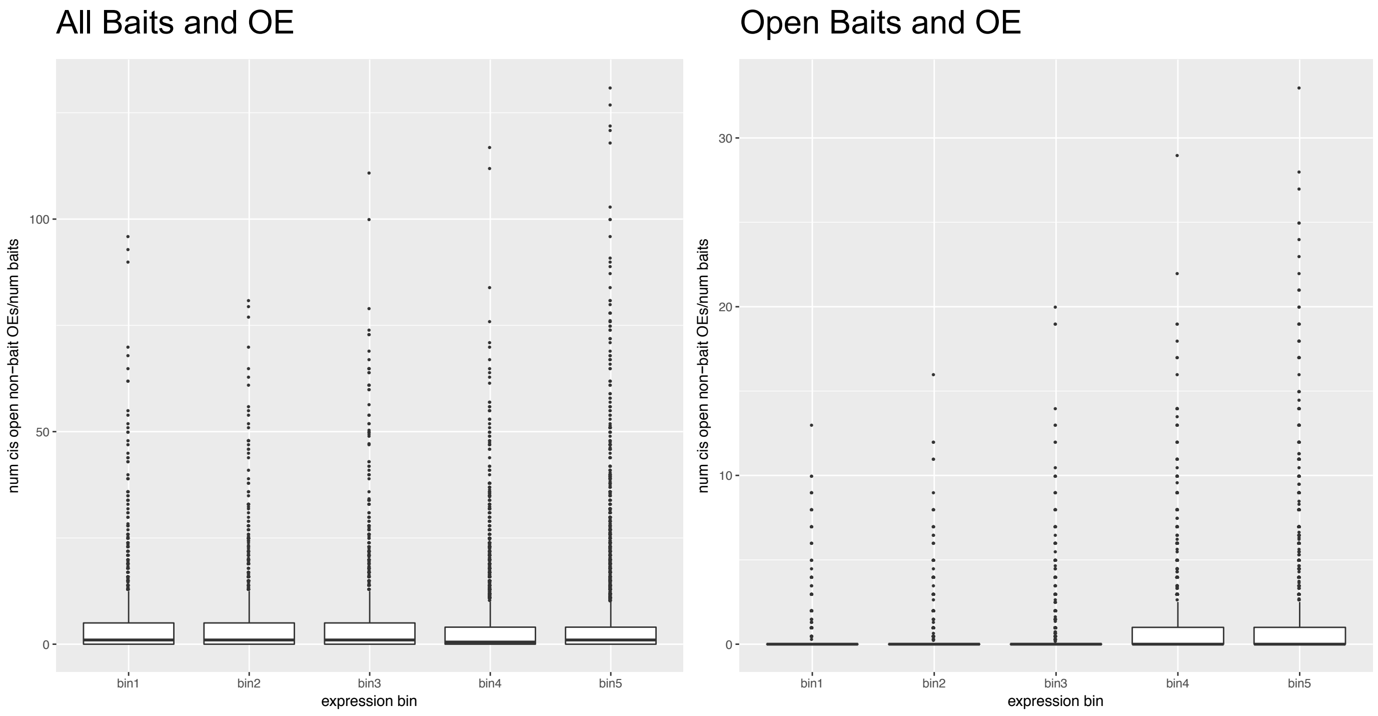

Correlation between number of 3D contacts per bait and gene expression. The number of contacts per bait correlates with gene expression, but only when considering open promoters and open PIR. Boxplots of the number of (cis) contacts per bait (at 4-fragment resolution; bait-to-bait interactions were excluded) for each quintile of expression of the baited gene(s). The horizontal line represents the median, the lower and upper hinges correspond to the 25th and 75th percentiles, and outliers (> 1.5 * IQR from the hinges, indicated by the whiskers) are plotted as single dots. Left: all baits and PIR. Right: open baits and open PIR only.

Supplementary Figure 3

A

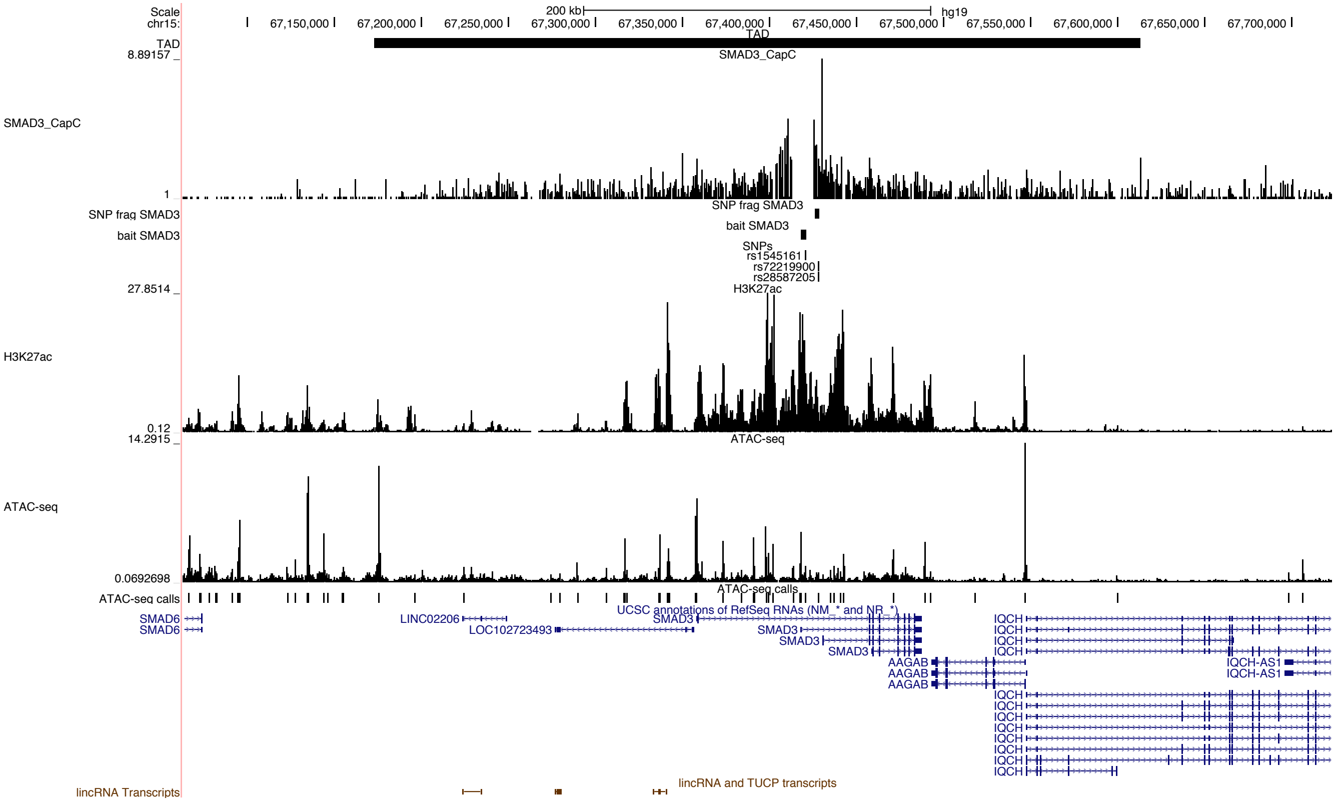

B

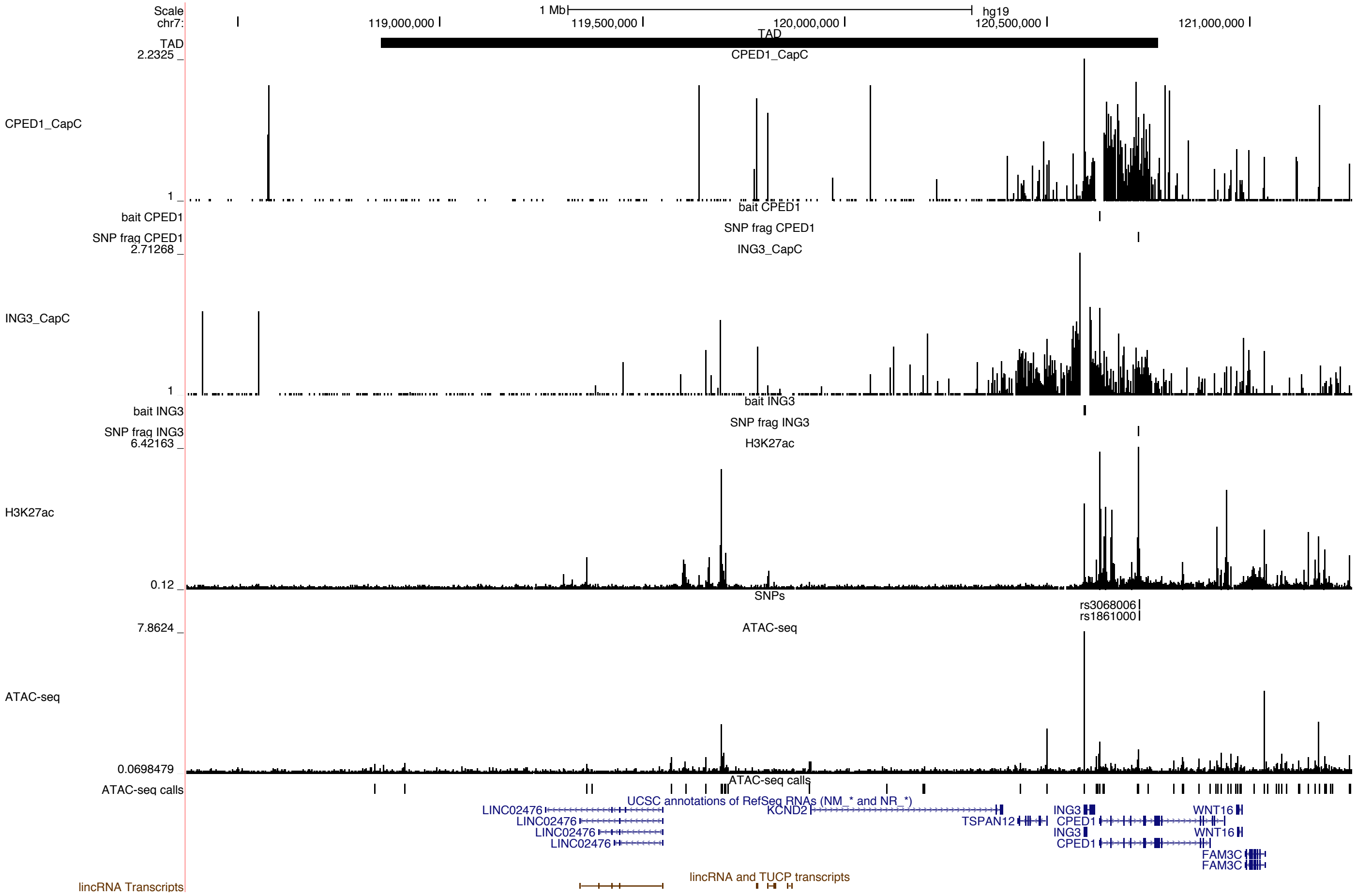

Scale
chr7:
1 Mb
hg19
119,000,000
119,500,000
120,000,000
120,500,000
121,000,000
TAD
CPED1_CapC
2.2325
1
bait CPED1
SNP frag CPED1
ING3_CapC
2.71268
bait ING3
SNP frag ING3
H3K27ac
6.42163
0.12
SNPs
rs3068006
rs1861000
ATAC-seq
7.8624
0.0698479
ATAC-seq calls
UCSC annotations of RefSeq RNAs (NM_* and NR_*)
LINC02476
KCND2
TSPAN12
ING3
CPED1
WNT16
FAM3C
lincRNA Transcripts
lincRNA and TUCP transcripts

C

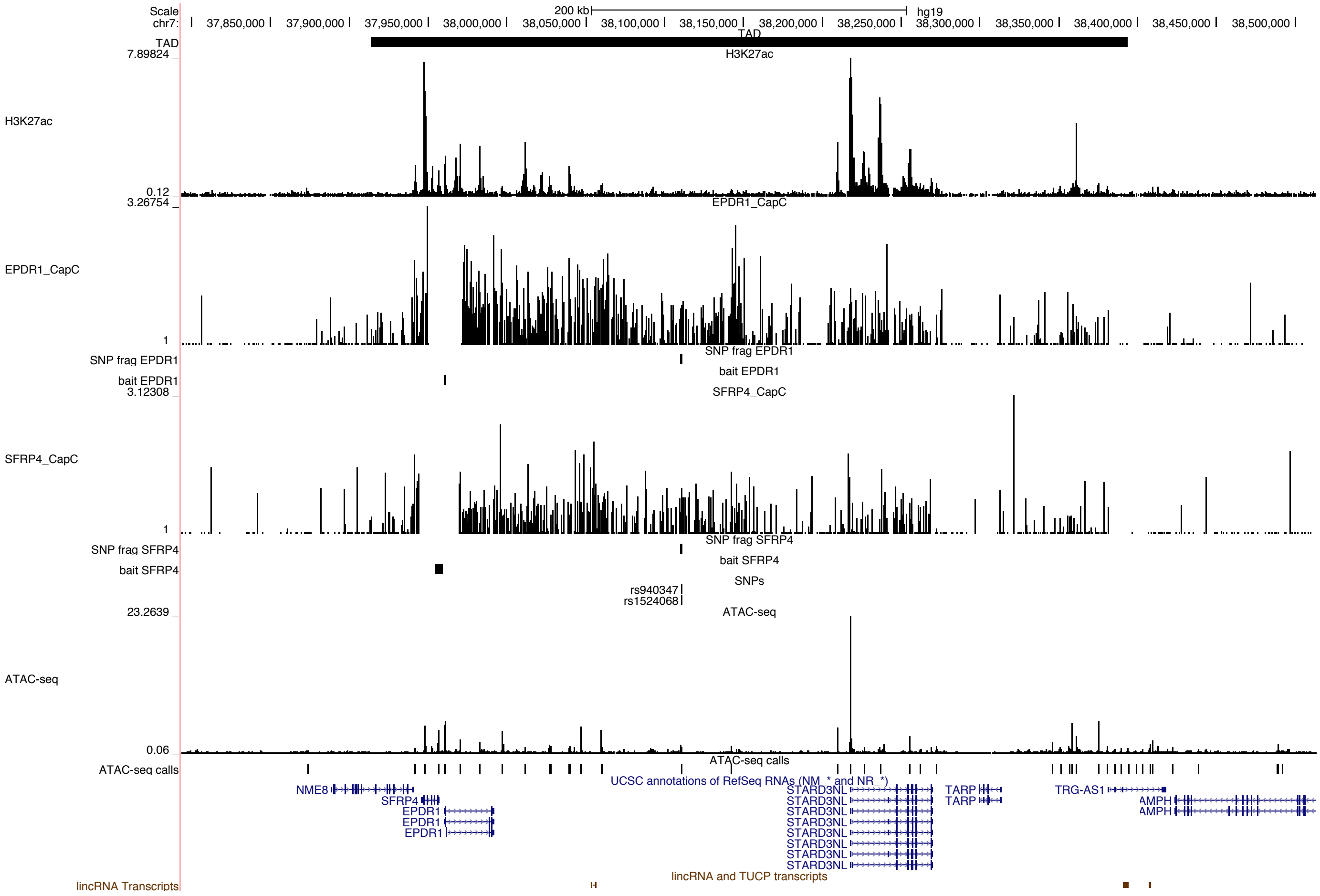
Scale
chr7:
200 kb
hg19
37,850,000
37,900,000
37,950,000
38,000,000
38,050,000
38,100,000
38,150,000
38,200,000
38,250,000
38,300,000
38,350,000
38,400,000
38,450,000
38,500,000
TAD
7.89824 _
H3K27ac
0.12
3.26754 _
EPDR1_CapC
1
SNP frag EPDR1
bait EPDR1
3.12308 _
SFRP4_CapC
1
SNP frag SFRP4
bait SFRP4
SNPs
rs940347
rs1524068
23.2639 _
ATAC-seq
0.06
ATAC-seq calls
UCSC annotations of RefSeq RNAs (NM_* and NR_*)
NME8
SFRP4
EPDR1
STARD3NL
TARP
TRG-AS1
AMPH
lincRNA Transcripts
lincRNA and TUCP transcripts

D

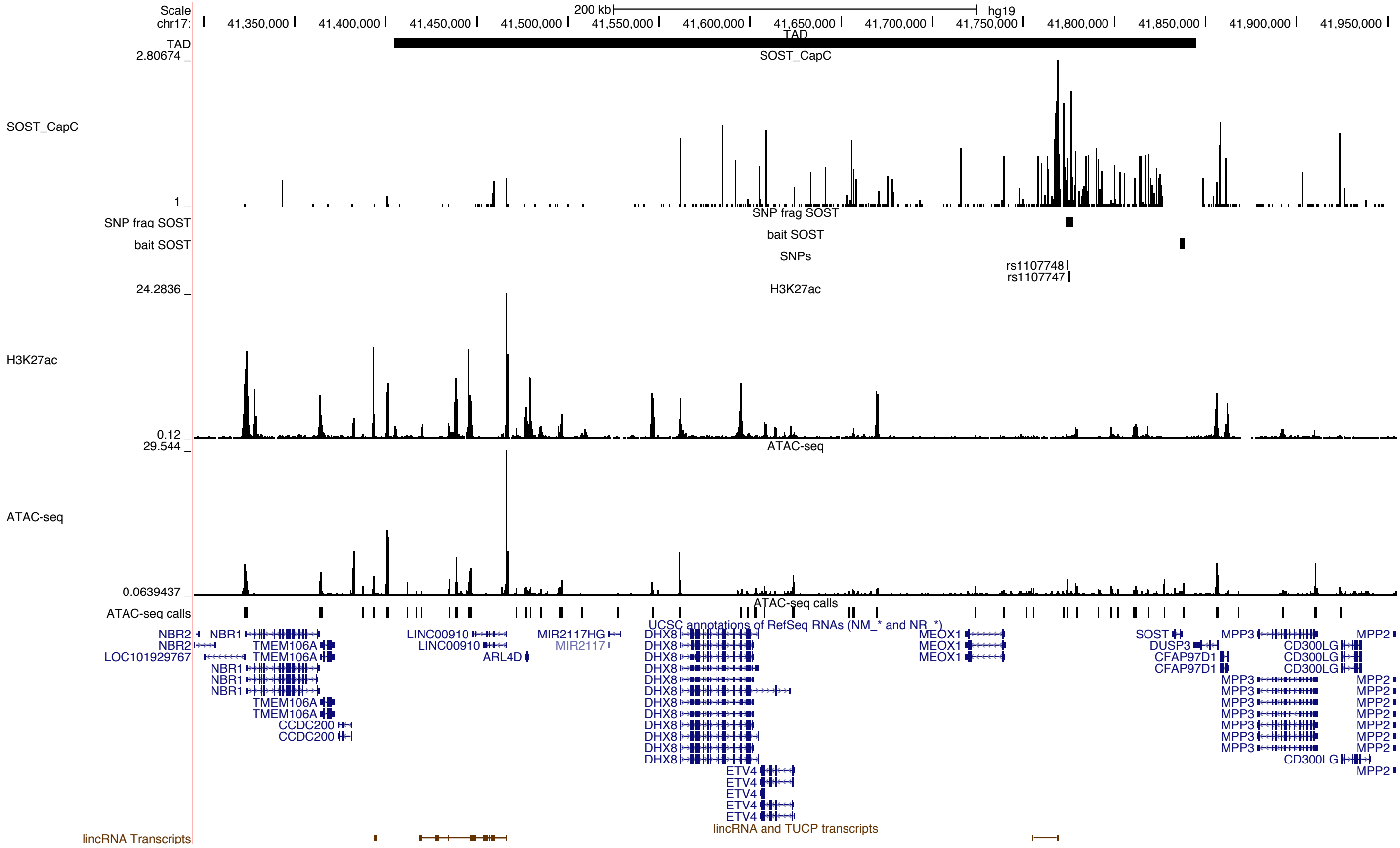

Capture C and ATAC-seq coverage tracks from hMSC-derived osteoblasts. Tracks are shown in the UCSC genome browser in BigWig format, from 3 and 9 replicates, respectively (see Supplementary Table 1 for details). ATAC-seq peak calls from our pipeline and H3K27ac ChIP-seq track from human primary osteoblasts from ENCODE (GEO accession GSM733739) are also shown in BED and bigWig format, respectively. SNP and bait fragments are indicated. (A) *SMAD3*, (B) *CPED1*, (C) *STARD3NL* and (D) *SOST*.

Supplementary Figure 4

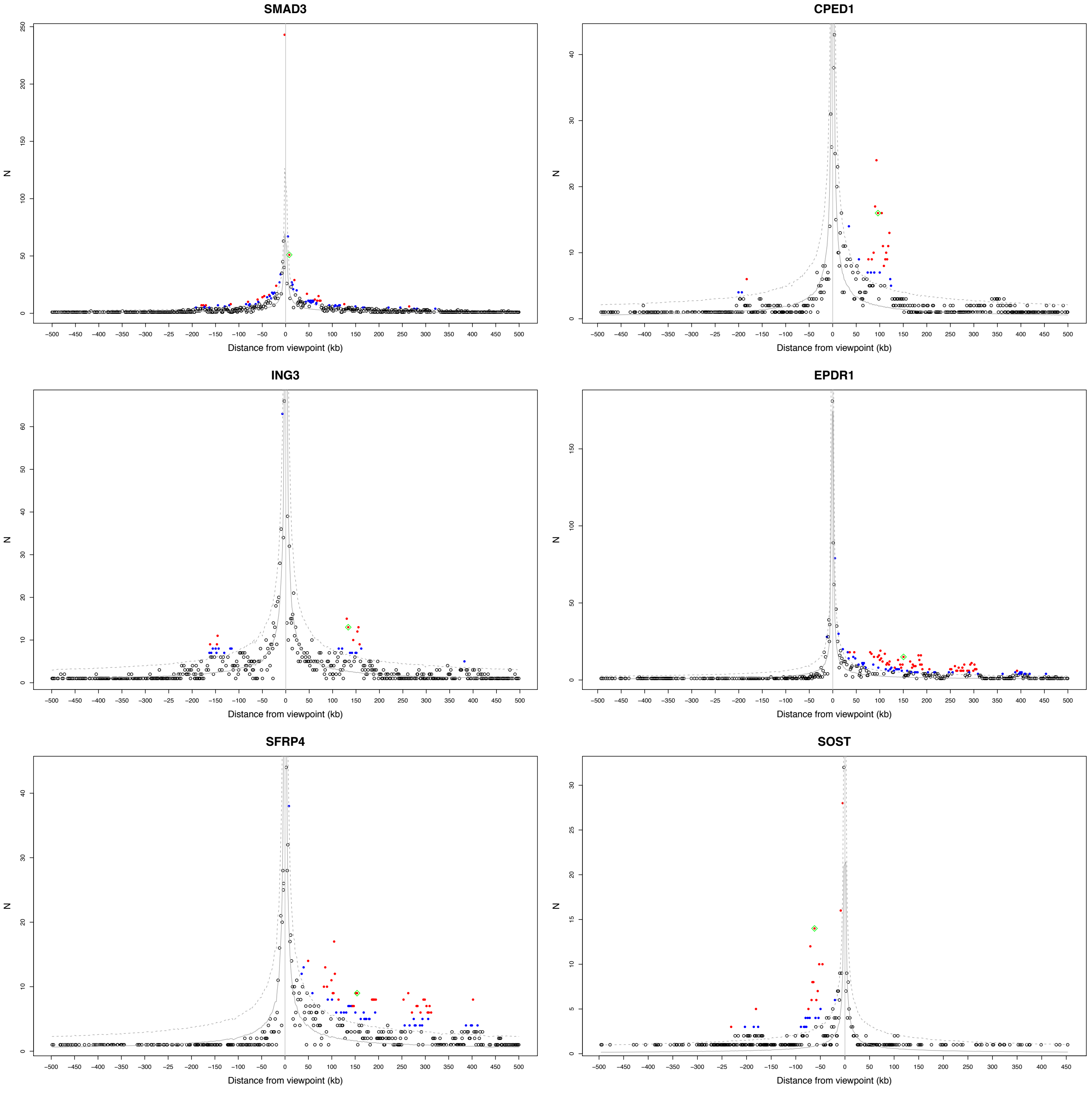

Examples of interactions called by CHiCAGO. Plots of the Capture C raw read counts versus linear distance from bait for the five loci analyzed more in depth in this study, plus *SOST* (as a positive control). Significant interactions (CHiCAGO score >5) are labelled in red, near-significant (CHiCAGO score >3) in blue. A region of 1Mb around the bait is shown. CHiCAGO-generated expected counts and their 95% confidence intervals are shown as solid and dashed grey lines, respectively. Data points representing significant interactions with regions containing GWAS proxy SNPs are highlighted with a green diamond.

Supplementary Figure 5

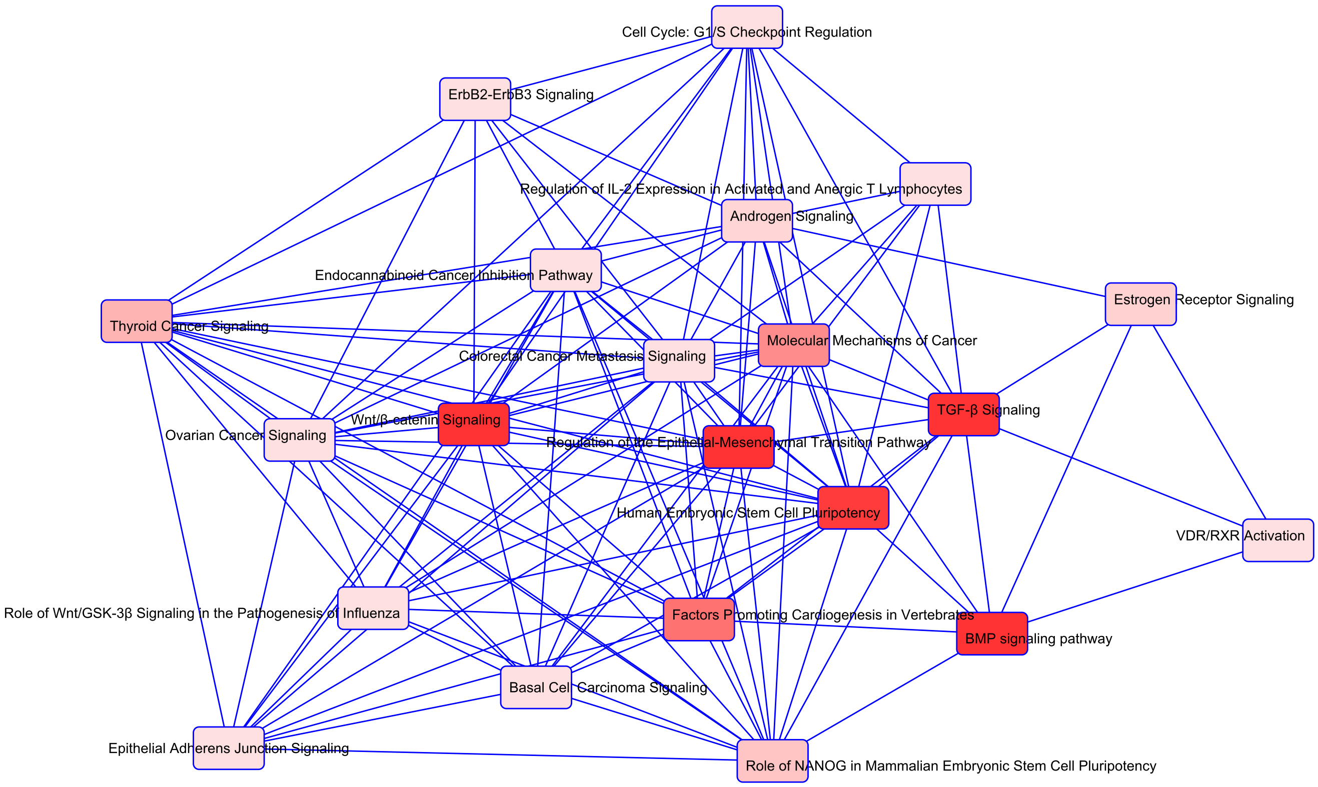

Canonical pathways enriched in implicated genes in osteoblasts. The genes were implicated by ATAC-seq plus Capture C in hMSC-derived osteoblasts using a r^2>0.4 threshold for proxy SNPs of 273 BMD GWAS loci. The network shows each pathway as a single "node" colored proportionally to the B-H multiple testing corrected p-value, where brighter red = more significant. A line connects any two pathways when they have at least two implicated genes in common between them. The analysis was performed using Ingenuity Pathway Analysis software.

Supplementary Figure 6

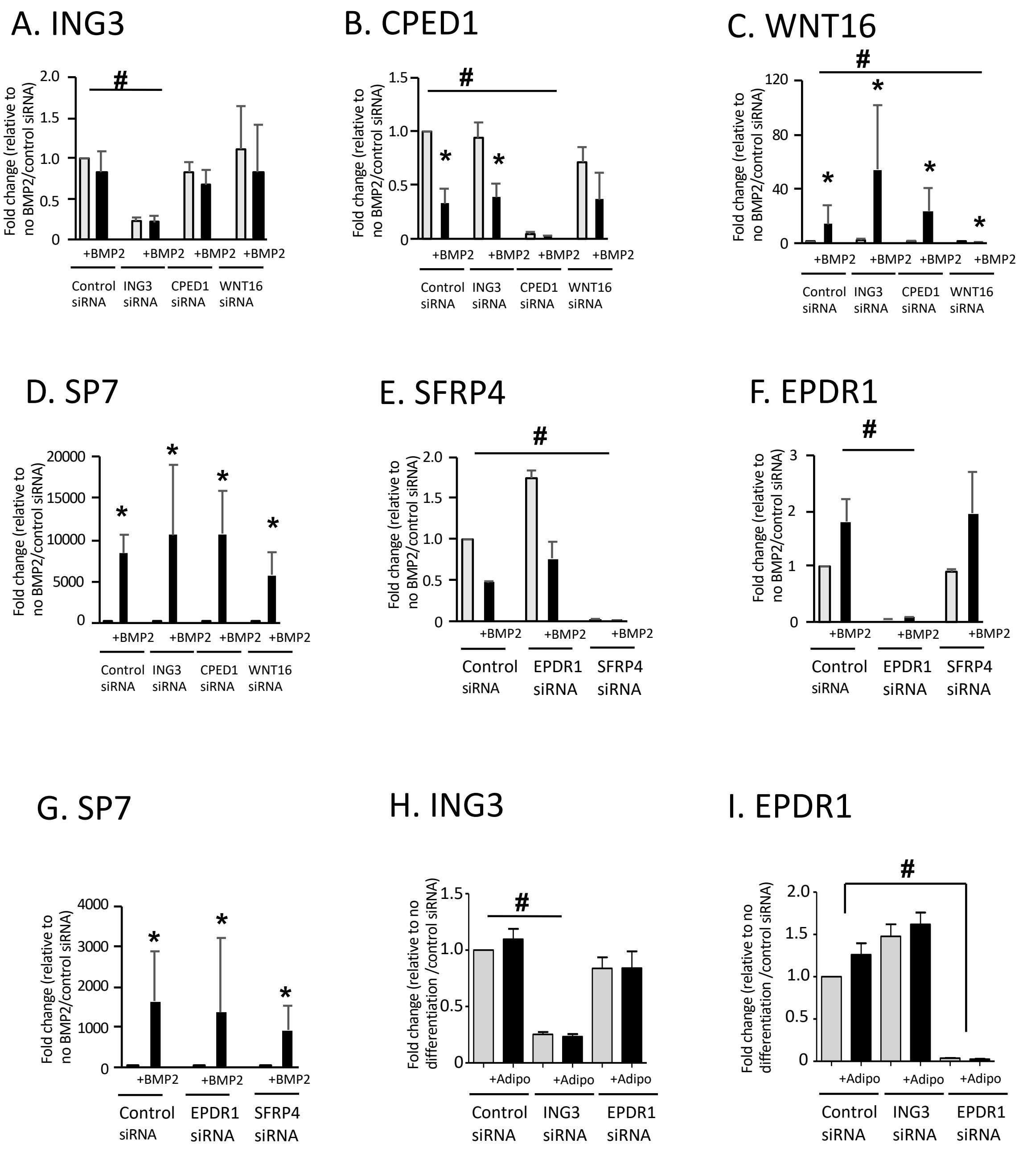

Proof of successful knockdown for the two GWAS loci analyzed. Quantitative gene expression in hMSC-derived osteoblasts (A-G) and adipocytes (H, I). (A-C) Knockdowns of *ING3*, *CPED1* and *WNT16* at the *CPED1* locus. (D) *SP7* expression when knocking down genes at the *CPED1* locus. (E-F) Knockdowns of *SFRP4* and *EPDR1* at the *STARD3NL* locus.

(G) *SP7* expression when knocking down genes at the *STARD3NL* locus. (H, I) Knockdowns of *ING3* and *EPDR1* in hMSC-derived adipocytes. Grey columns = No treatment; Black columns = differentiation treatment (BMP or adipogenic differentiation). Columns = mean. Error bars = Standard deviation. n=4 (for *ING3*, *CPED1* and *WNT16* siRNA with BMP treatment), n=3 (for *SFRP4* and *EPDR1* siRNA with BMP2 treatment) and n=4 (for *ING3* and *EPDR1* siRNA with adipogenic differentiation) unique donor lines. *, $p<0.05$ comparing No treatment to treatment for each siRNA. #, $p<0.05$ comparing control siRNA to siRNA for gene of interest (2-way homoscedastic Student's t-tests). Source data are provided as a Source Data file.

Supplementary Figure 7

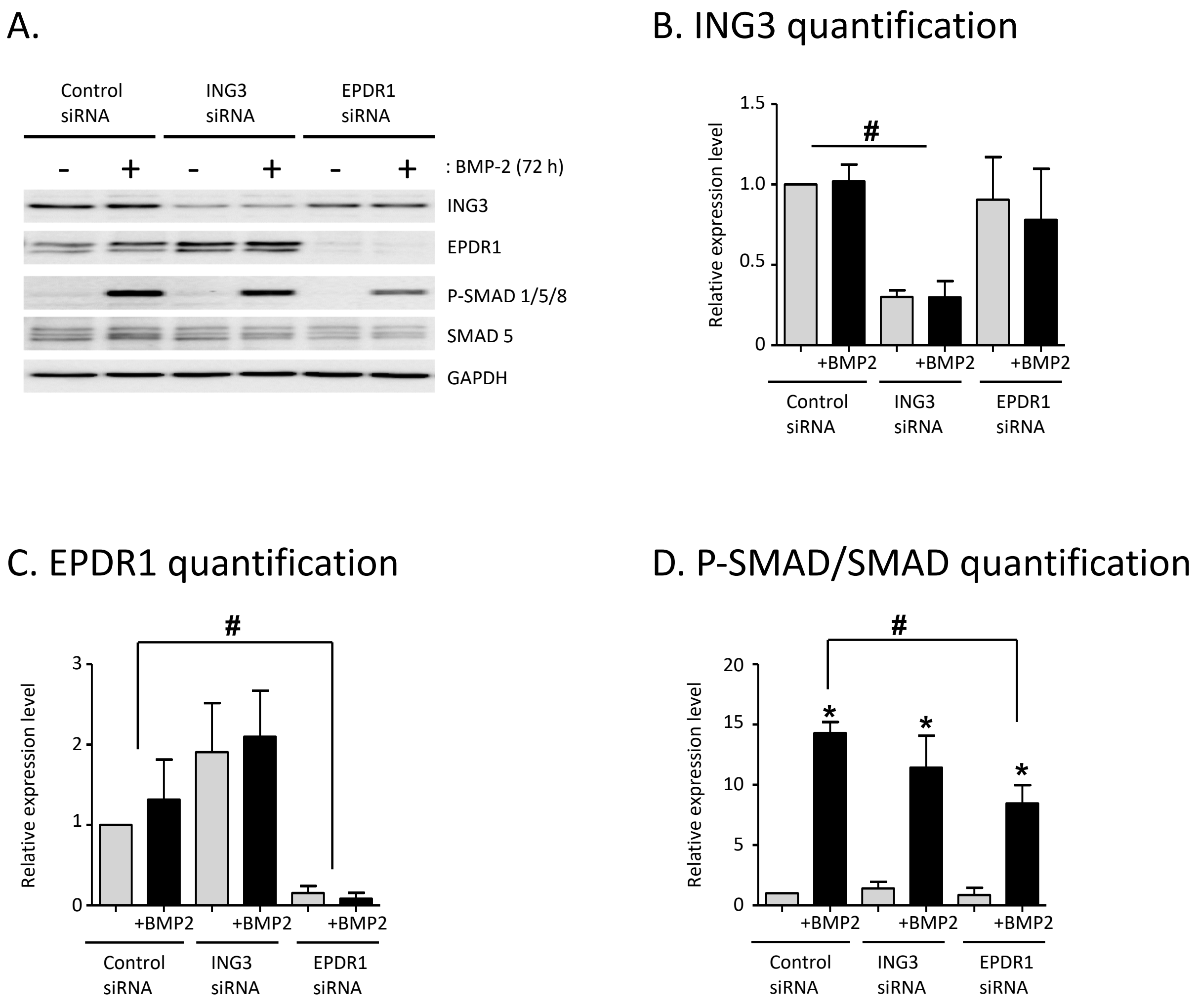

ING3 and EPDR1 siRNA decreases protein levels of respective targets. (A) Total cell lysates from *ING3* and *EPDR1* siRNA transfected cells co- stimulated with BMP2 for 72 h were processed for immunoblot analysis with antibodies as indicated. A representative result of 4 different hMSC donor line is shown. (B-D) Relative band intensities from each hMSC donor cell line were combined and presented as relative expression compared to control siRNA transfected sample. Grey columns = No BMP treatment; Black columns = BMP treatment. Columns = mean. Error bars = Standard deviation. n=4 unique donor lines. *, $p<0.05$ comparing No treatment to BMP treatment for each siRNA. #, $p<0.05$ comparing control siRNA to siRNA

for gene of interest (2-way homoscedastic Student's t-tests). Source data are provided as a Source Data file.
