# Supplementary material for: Genome-scale Capture C promoter interactions implicate effector genes at GWAS loci for bone mineral density
Source: Nat Commun. 2019 Mar 19;10:1260. doi: 10.1038/s41467-019-09302-x (PMC6425012; doi:10.1038/s41467-019-09302-x)
Supplement: Supplementary file 2 — Description of Additional Supplementary Files [file 41467_2019_9302_MOESM2_ESM.pdf]

Description of Additional Supplementary Files

Supplementary Data 1. BMD loci investigated in this study (DEXA and heel ultrasound); ATAC-seq ENCODE IDR conservative narrowPeaks from 9 replicates of hMSC-derived osteoblasts; open proxies of the BMD GWAS sentinel SNPs at r^2>=0.8 and r^2>=0.4.

Supplementary Data 2. Summary statistics from HiCUP pre-processing of Capture C data from 3 replicates of hMSC-derived osteoblasts (from 3 independent donors) and 3 replicates of HepG2 cells.

Supplementary Data 3. RNA-seq expression data of hMSC-derived osteoblasts from 3 independent donors. Fpkm and percentile of expression are reported for all genes and ATAC-seq plus Capture C implicated genes. Implicated genes with zero expression are also reported.

Supplementary Data 4. Capture C interaction data from hMSC-derived osteoblasts from 3 independent donors. Significant calls from CHiCAGO with CHiCAGO score >=5 are reported for the BMD GWAS loci investigated (DEXA and heel ultrasound), using open proxies with r^2>=0.8 and r^2>=0.4.

Supplementary Data 5. Capture C interaction data from 3 replicates of HepG2 cells. Significant calls from CHiCAGO with CHiCAGO score >=5 are reported for the 273 BMD GWAS loci investigated (DEXA and heel ultrasound), using open proxies with r^2>=0.8.

Supplementary Data 6. Capture C interaction data from hMSC-derived adipocytes from 3 independent donors. Significant calls from CHiCAGO with CHiCAGO score >=5 are reported for the 3 BMD loci investigated more in depth in this study (*CPED1*, *STARD3NL* and *SMAD3*).

SOURCE DATA

Source data for Figures 2 and 3 and Supplementary Figures 6 and 7.

Figure 2: quantitative gene expression of key osteogenesis markers plus Alk Phos and Alizarin Red staining in hMSC-derived osteoblasts transfected with ING3, CPED1, WNT16, EPDR1 and SFRP4 siRNA.
Figure 3: quantification of Oil Red O -stained lipid droplets and quantitative gene expression for C/EBP alpha and PPAR gamma in hMSC-derived adipocytes transfected with ING3 and EPDR1 siRNA.
Supplementary Figure 6: quantitative gene expression for ING3, CPED1, WNT16 and SP7 in hMSC-derived osteoblasts transfected with ING3, CPED1, WNT16, EPDR1 and SFRP4 siRNA. Quantitative gene expression for ING3 and EPDR1 in hMSC-derived adipocytes transfected with ING3 and EPDR1 siRNA.
Supplementary Figure 7: quantification of ING3, EPDR1 and p-SMAD/SMAD protein levels from Western blots from hMSC-derived osteoblasts transfected with ING3 and EPDR1 siRNA.
